# Adaptation of the Critical Care Family Need Inventory to the Turkish population and its psychometric properties

**DOI:** 10.7717/peerj.1208

**Published:** 2015-08-20

**Authors:** Sibel Büyükçoban, Meltem Çiçeklioğlu, Nilüfer Demiral Yılmaz, M. Murat Civaner

**Affiliations:** 1 Department of Anesthesiology and Reanimation, Nazilli State Hospital, Nazilli, Aydin, Turkey; 2 Department of Public Health, Ege University School of Medicine, Bornova, Izmir, Turkey; 3 Department of Medical Education, Ege University School of Medicine, Bornova, Izmir, Turkey; 4 Department of Medical Ethics, Uludag University School of Medicine, Gorukle, Bursa, Turkey

**Keywords:** Intensive care, Patient satisfaction, Patient relatives, Patient rights, Critical Care Family Needs Inventory, Psychometric properties

## Abstract

In the complex environment of intensive care units, needs of patients’ relatives might be seen as the lowest priority. On the other hand, because of their patients’ critical and often uncertain conditions, stress levels of relatives are quite high. This study aims to adapt the Critical Care Family Need Inventory, which assesses the needs of patients’ relatives, for use with the Turkish-speaking population and to assess psychometric properties of the resulting inventory. The study was conducted in a state hospital with the participation of 191 critical care patient relatives. Content validity was assessed by expert opinions, and construct validity was examined by exploratory factor analysis (EFA). Cronbach’s alpha coefficient was used to determine internal consistency. The translated inventory has a content validity ratio higher than the minimum acceptable level. Its construct validity was established by the EFA. Cronbach’s alpha coefficient for the entire scale was 0.93 and higher than 0.80 for subscales, thus demonstrating the translated version’s reliability. The Turkish adaptation appropriately reflects all dimensions of needs in the original CCFNI, and its psychometric properties were acceptable. The revised tool could be useful for helping critical care healthcare workers provide services in a holistic approach and for policymakers to improve quality of service.

## Introduction

In intensive care units (ICUs), which mostly serve patients with severe health problems, healthcare workers need to make decisions that are simultaneously swift and correct. In the complex nature of this environment, the needs of the patient’s relatives might be seen as the lowest priority (Mendonca & Warren, 1998). On the other hand, because of the patient’s critical and often uncertain condition, the family’s stress levels are often quite high (Ozgursoy & Akyol, 2008). Meeting relatives’ needs—to be informed clearly and honestly, for example—might reduce their stress (Kutlu, 2000; Price et al., 1991; Siddiqui, Sheikh & Kamal, 2011). Physicians and especially critical care nurses are in the best position to help relatives (Leske, 1986; Ozgursoy & Akyol, 2008). However, relevant literature studies have shown that the specific needs and concerns of relatives of critical care patients are not properly met (Curry, 1995; Kleinpell & Powers, 1992), and the most cited reason for not meeting the needs is misjudgement of the importance of those needs (Johnson et al., 1995; Leung, Chien & Mackenzie, 2000). Thus, determining and understanding the dimensions of relatives’ needs seems the first step toward improving quality of service, and also for exercising a major patient right—to be informed in a thorough and timely manner.

Molter (1979), who conducted one of the earliest studies on the needs of critical care patients’ relatives, constructed a needs list. Leske modified this list and titled it Critical Care Family Need Inventory (CCFNI) (Leske, 1991), defined its aim as determining the level of importance of relatives’ needs and developing a tool for clinics to measure categories of family needs. The inventory has been adapted into various languages and cultures, and validity and reliability studies performed accordingly (Bandari et al., 2014; Bijttebier et al., 2000; Chien, Ip & Lee, 2005; Coutu-Wakulczyk & Chartier, 1990; Macey & Bouman, 1991; Sungeun, 2008). This inventory has been used in many subsequent studies (Lee & Lau, 2003; Leung, Chien & Mackenzie, 2000; O’Malley et al., 1991; Quinn, Redmond & Begley, 1996). In fact, two previous studies have translated CCFNI into Turkish. Conducted by Unver (1998), the first study assessed reliability by Cronbach’s alpha coefficient, while Toros (2003), in addition to reliability analysis, assessed content validity by Kendall’s coefficient of concordance. However, these two studies basically assumed that the inventory is unidimensional and that all 45 items measure the same “need” dimension. Yet, CCFNI items are quite heterogeneous. The constructed validity of the original inventory, which comprised five subscales, was not assessed in those two studies. Furthermore, conducting comprehensive translation processes is not sufficient for adapting an inventory into a different language (Gjersing, Caplehorn & Clausen, 2010), and therefore all reliability and validity analyses must be redone (Artino et al., 2014). To use an inventory developed for a specific culture in another language or culture, its psycholinguistic and psychometric properties must be (re)assessed (Ægisdóttir, Gerstein & Çinarbaş, 2008). This issue was not properly addressed by earlier translations.

This study was designed to adapt the CCFNI to the Turkish speaking population. It addition, it aimed to estimate language validity, content validity, construct validity, and discriminant validity to support construct validity and reliability, considering CCFNI subscales as well. Overall, this study aims to provide an inventory for healthcare workers and policymakers to use for understanding critical care patient relatives’ needs and thus improving healthcare.

## Methods

This study was methodologically designed to translate CCFNI into Turkish and then to determine its validity and reliability.

### Original inventory

Molter (1979) has acknowledged that the content validity of the needs list she has developed was limited, and has given no information on its reliability. Molter’s needs list, modified by Leske (1991) and titled CCFNI, now assesses needs of critical care patients’ relatives with 45 items. It uses a four-point Likert scale *(1: not important; 2: slightly important; 3: important; 4: very important).* In the construct validity analysis, five subscales were defined: *Support* (n: 15), *Comfort* (n: 6), *Information* (n: 8), *Proximity* (n: 9), and *Assurance* (n: 7). Calculated for internal consistency, Cronbach’s alpha coefficients lie between 0.61 and 0.88 for subscales, and for the entire inventory the Cronbach’s alpha coefficient is 0.92 (Leske, 1991).

### Translation into Turkish

In adapting CCFNI to Turkish, it was first translated using a four-step methodology (Sousa & Rojjanasrirat, 2011).

• *Forward translation*: Two physicians with a good command over English and a linguistics expert each independently translated the original inventory from English to Turkish.
• *Reconciliation*: The three translators and researchers compared the forward versions with the original inventory and reconciled their differences.
• *Back translation*: A professional translator with a good command over both Turkish and English translated the reconciled version from Turkish back to English.
• *Comparison:* As a last step, the original and back-translated versions were reevaluated. The back-translation was compared with the original instrument to determine whether any differences between the English and Turkish inventories in meaning and concept coherence. After the necessary corrections, the inventory assumed its final target language version.

This approach provided concept and language equivalence; therefore, an agreement on the inventory’s final translation was achieved.

### Validity and reliability

#### Content validity

After the translation process was completed, experts assessed the Turkish version’s content validity. Ten experts, four anaesthesiology specialists, and six intensive care nurses, were asked to assess the items’ content, meaning, and comprehensibility using terms proposed as *inappropriate; appropriate to some extent—item should be revised; appropriate—minor changes required*; and *quite appropriate* (Gozum & Aksayan, 2003; McKenzie et al., 1999). The assessments *appropriate—minor changes required* and *quite appropriate* were accepted as *appropriate* overall, and content validity ratio (CVR) was calculated accordingly (Lawshe, 1975). Experts’ suggestions were also solicited, and these were used to revise items. After that step, to assess its comprehensibility, the Turkish CCFNI version was reviewed by a panel comprising eight people: two patient relatives, two critical care nurses, two physicians working in intensive care, and two researchers. Revisions suggested by the abovementioned ten experts were evaluated and rephrased by the panel to increase the inventory’s comprehensibility while maintaining its meaning.

#### Pre-testing

The inventory, which was improved by expert opinions, was pre-tested through participation by 15 volunteer patient relatives. These volunteers evaluated inventory items for readability, comprehensibility, sentence length, clearness and clarity of meaning to ready the inventory for implementation. After pre-testing, no changes to the content of the Turkish version were required.

#### Implementation

The CCFNI Turkish version was implemented at Nazilli State Hospital in the city of Aydin, Turkey. The hospital serves a population of 350,000, and also a substantial amount of travelers since Aydin is located near a busy highway between megalopolis cities like Istanbul and holiday resorts in south Turkey. As Chien, Ip & Lee (2005) quoted, Stevens suggests that at least 200 cases are required for exploratory factor analysis to ensure the reliability of the factor structure. A convenience sampling of 204 family members of critical care patients admitted to ICUs from September to December 2012. However, eight relatives withdrew from the study, and five failed to complete the inventory. Thus, data were collected from 191 participants who were relatives of patients older than 18, with educational levels from 6th grade to university graduation, within 24–72 h of the patient’s hospitalization. In this study, the spouse and blood-related family members are considered relatives. After being informed about the study and voluntarily consenting to participate, relatives completed the self-reported inventory in 10–15 min.

#### Data analysis

Construct validity was examined with exploratory factor analysis (EFA) to explore a possible different factor structure of the inventory in the Turkish-speaking population (Buyukozturk, 2004). Sampling adequacy for factor analysis was measured by Kaiser-Meyer-Olkin (KMO) and Bartlett’s test of sphericity. Principal components analysis and Varimax rotation were used in factor analysis; eigenvalues, percentage of total variance explained, scree plot, and factor loadings were examined together. The factor in the steep curve of scree plot, before the first point that started the flat line trend, was identified as a “significant factor,” in other words, the number of factors with eigenvalues higher than 2 (Buyukozturk, 2004).

Discriminant validity analysis was conducted by student *t*-test and effect size to support construct validity. The relationship between variables of the patient’s diagnosis, relative’s gender, degree of relatedness, and scores obtained from the entire inventory and its subscales were evaluated.

Reliability analysis was performed by calculating Cronbach’s alpha coefficients of the entire inventory, its subscales, and item-total correlation coefficients.

Significance level was set at 0.05 for all tests, and the confidence interval was 95%. Statistical analysis was performed using PASW statistics for Windows (SPSS, Inc., IBM) version 21.0.

#### Ethical considerations

Written permission to adapt the inventory for the Turkish population was obtained from both Nancy C. Molter and Jane S. Leske. Official permission of Aydin Health Directorate and approval of Ege University School of Medicine’s Research Ethics Committee were granted (Approval number 12-09/12). Participants were informed of the study’s aims and methods and of their ability to leave the study anytime they wanted without any consequence to the patient’s care. They were enrolled in the study only after their consent was obtained.

## Results

### Sample characteristics

The mean age of the patient relatives was 43.91 years (SD, 12.54 years; range, 18–74 years), and 54.0% were women. The relatives were adult children (40.8%), parents (16.7%), spouses (8.9%), and other relatives (32.9%). The patients’ mean age was 66.14 years (SD, 17.60 years; range, 18–96 years), and 57.4% of patients were men. Of all patients, 16.8% were receiving intensive care because of trauma, while others were being treated for internal diseases.

### Validity of the Turkish inventory

#### Content validity

On rating the translation’s appropriateness by the ten health professionals, only two items, *To have directions as to what to do at the bedside* and *To have a telephone near the waiting room* were considered by more than two raters as inappropriately translated. They were modified to the following: *To have a list of what to do when visiting my patient* and *To have a telephone I can use near the waiting room*. According to the expert assessments, the items’ CVR were between 0.80 and 1.00. These values were compared to the table of minimum CVR proposed by Lawshe (1975). As defined in this table, minimum CVR value for ten experts was 0.62. Therefore, the experts accepted all the inventory’s items. In the last step of content validity, a panel of eight people reviewed the inventory and suggested revision for only one item: *To know which staff member could give what type of information* was modified to *To know which staff member (physician, nurse, medical secretary) could give what type of information*.

#### Construct validity

KMO and Bartlett’s test of sphericity were used to measure sampling adequacy for factor analysis. The values of KMO (0.87) and Bartlett’s test of sphericity (*χ*^2^: 4510.8; *p* < 0.00) were considered significant, and it was accepted that the data was appropriate for factor analysis. Principal component analysis explained the three components with eigenvalues exceeding 2.0 in the unrotated matrix. Varimax rotation was performed on all 45 items. Item loadings above 0.30 and items within 0.10 of the highest loading were prohibited. The following five items failed to meet these criteria and were omitted: *To have visiting hours changed for special conditions; To have the waiting room near the patient; To have explanations given that are understandable; To have someone to help with financial problems;* and *To have visiting hours start on time*.

This three-subscale factorial structure explains 43.66% of total variance. Items’ factor loadings varied from 0.34 to 0.79. Table 1 displays eigenvalues, percentage of total variance explained, and factor loadings.

**Table 1:** Factor structure of the Turkish CCFNI.

Factors	Eigenvalues	Percentage of total variance explained	Cumulative percentage of total variance explained	Factor loadings
F1	11.8	18.50	18.50	0.78–0.37
F2	3.65	12.90	31.41	0.73–0.34
F3	2.01	12.25	43.66	0.79–0.42

Consequently, the revised inventory comprised 40 items, and the subscales’ names were rephrased because the Turkish version had a three-factor structure, i.e., *Need for support and comfort; Need for proximity and assurance; need for information*. The CCFNI Turkish version’s three factor structure differs from the original inventory in item categorization. Table 2 shows which item belongs to which factor in Leske’s result.

**Table 2:** Item factor loadings, Cronbach’s alpha and item-total correlation coefficients.

Item number *(Original inventory factor)*	Items	Factor loading			Item-total correlation
		F1	F2	F3	
**Support/comfort** *(Cronbach’s *α: 0.92)**					
33 (*S*)	To be alone at any time	0.78			0.56
27 (*S*)	To have someone be concerned about with your health	0.69			0.61
26 (*S*)	To have another person with you when visiting the critical care unit	0.68			0.64
18 (*S*)	To have a place to be alone while in the hospital	0.68			0.60
37 (*I*)	To be told about chaplain services	0.66			0.48
30 (*S*)	To feel it is all right to cry	0.65			0.53
24 (*S*)	To have a pastor visit	0.64			0.48
29 (*P*)	To talk to the same nurse every day	0.62			0.62
20 (*C*)	To have comfortable furniture in the waiting room	0.60			0.58
38 (*I*)	To help with the patient’s physical care	0.58			0.63
34 (*S*)	To be told about someone to help with family problems	0.57			0.58
32 (*C*)	To have a bathroom near the waiting room	0.55			0.56
8 (*C*)	To have good food available in the hospital	0.53			0.53
10 (*P*)	To visit at any time	0.53			0.58
23 (*C*)	To have a telephone near the waiting room	0.49			0.55
44 (*P*)	To see the patient frequently	0.47			0.62
21 (*C*)	To feel accepted by the hospital staff	0.47			0.65
31 (*S*)	To be told about other people that could help with problems	0.47			0.56
12 (*S*)	To have friends nearby for support	0.44			0.43
7 (*S*)	To talk about feelings about what has happened	0.37			0.42
**Assurance/proximity** *(Cronbach’s* *α: 0.83)*					
43 (*A*)	To know specific facts concerning the patient’s progress		0.73		0.38
42 (*A*)	To feel that the hospital personnel cares about the patient				
5 (*A*)	To have questions answered honestly		0.63		0.28
41 (*P*)	To receive information about the patient at least once a day		0.62		0.35
14 (*A*)	To feel there is hope		0.62		0.34
28 (*C*)	To be assured it is all right to leave the hospital for a while		0.62		0.44
17 (*A*)	To be assured that the best care possible is being given to the patient		0.61		0.46
11 (*I*)	To know which staff members could give what type of information		0.56		0.49
3 (*I*)	To talk to the doctor every day		0.53		0.32
9 (*S*)	To have directions as to what to do at the bedside		0.52		0.41
2 (*S*)	To have explanations of the environment before going into the critical care unit for the first time		0.34		0.37
**Information** *(Cronbach’s* *α: 0.84)*					
16 (*I*)	To know how the patient is being treated medically			0.79	0.59
19 (*I*)	To know exactly what is being done for the patient			0.78	0.53
15 (*I*)	To know about types of staff members taking care of the patient			0.64	0.57
25 (*S*)	To talk about the possibility of the patient’s death			0.59	0.56
13 (*I*)	To know why things were done for the patient			0.57	0.49
39 (*P*)	To be told about transfer plans while they are being made			0.54	0.46
40 (*P*)	To be called at home about changes in the patient’s condition			0.52	0.39
4 (*I*)	To have a specific person to call at the hospital when unable to visit			0.51	0.45
36 (*P*)	To have visiting hours start on time			0.42	0.49

Completion of the discriminant validity analysis determined that the inventory was able to discriminate among participants according to the following independent variables: patient’s diagnosis, patient relative’s gender, and degree of relatedness (Table 3).

**Table 3:** Distribution of subscale scores in terms of independent variables.

Variables	Total inventory	Support/comfort	Proximity/assurance	Information
Patient relative’s gender (Woman > man)	*P*^a^: 0.041 Effect size: 0.29	*P*^a^: 0.144	*P*^a^: 0.003 Effect size: 0.41	*P*^a^: 0.086
Degree of relatedness (Others > parents)	*P*^a^: 0.000 Effect size: 1.31	*P*^a^: 0.000 Effect size: 1.17	*P*^a^: 0.000 Effect size: 1.00	*P*^a^: 0.000 Effect size: 1.13
Patient’s diagnosis (Others > trauma/post-op)	*P*^a^: 0.000 Effect size: 0.71	*P*^a^: 0.000 Effect size: 0.87	*P*^a^: 0.008 Effect size: 0.51	*P*^a^: 0.01 Effect size: 0.47

**Notes.**

a Student’s *t* test

Effect size (Mean 1 − Mean 2)/Total standard deviation Effect size assessment criterion: 0.20 small 0.50 medium 0.80 large (Cohen, 1988)

We discovered that patients hospitalized because of internal diseases, women relatives, and relatives excluding parents had higher scores compared to others. The *need for support and comfort* and the *need for information* subscales were sensitive to patients’ diagnoses and degree of relatedness, while the *need for proximity and assurance* subscale was sensitive to the patient relative’s gender, degree of relatedness, and patient diagnosis.

### Reliability of the Turkish inventory

#### Internal consistency

Cronbach alpha coefficient calculated for the inventory’s Turkish version was 0.93 for the entire scale and from 0.83 to 0.92 for the three subscales (Table 2).

#### Item-total correlation

It was found that item-total correlation coefficient, which was calculated to determine the relationship of items with total score, lay between 0.28 and 0.65 for every item. The only item that fell below the 0.30 criterion was: *To have questions answered honestly* (item-total correlation of 0.28) (Table 2).

## Discussion

This study was designed, first, to adapt the CCFNI to the Turkish population and, second, to explore its content validity, construct validity, and reliability.

### Validity

After the translation process, the translated items in the Turkish version correspond to the original inventory’s items. This showed that language validity was ensured. In addition, CVR value calculated from expert opinions showed that all items complied with the inventory’s aim, so the translated inventory’s content validity was established. In the Toros (2003) study, correlation among expert opinions was low (Kendall’s coefficient w: 0.1343; *p* ≥ 0.05). Thus, it is possible to say that the version developed in this study adequately reflected the terms and concepts of CCFNI.

The Turkish inventory’s construct validity was established well enough with this study. Two kinds of diverseness emerged with factor analysis: five original items were omitted because of overlap, and the five-factor structure originally defined by Leske was modified to three factors. This three-factor structure explains 43.66% of total variance; in other words, the Turkish version’s factorial structure is acceptable since 40%–60% is the interval of acceptance for multifactorial patterns (Cokluk, Sekercioglu & Buyukozturk, 2010). The original five-factor structure of changed in both French (Coutu-Wakulczyk & Chartier, 1990) and Dutch adaptations: The items spread under quite different factors while retaining the five-factor structure (Bijttebier et al., 2000). Thus, it is possible to say that the three-factor structure is not inadequate because the subscales might vary with respect to different languages and cultures. Notably, future studies with larger samples would be valuable in further assessing the Turkish inventory’s factor structure.

Discriminant validity analysis revealed significant differences among scores obtained with respect to patient’s diagnosis, patient relative’s gender, and degree of relatedness. This indicates that the Turkish inventory demonstrating differences among participants according to some variables refers to its discrimination strength. This also supports construct validity. Women relatives, relatives excluding parents, and patients hospitalized because of internal diseases had higher scores compared to others. Previous studies have suggested that female relatives generally report higher need levels than male relatives (Coutu-Wakulczyk & Chartier, 1990; Price et al., 1991; Rukholm et al., 1991; Tekinsoy, 2005). In the study by Bijttebier et al. (2000), women relatives scored higher in all subscales except *Information*. In this study, the scores of patients’ parents were relatively low in all subscales. Tekinsoy (2005) also found that parents had lower needs scores in *Support* and *Comfort* subscales. The patient’s diagnosis was also discriminatory both in total inventory and its subscales. Needs of relatives whose patients have internal diseases are higher than other relatives’ needs. Similarly, Wong found that medical patients’ family members rated the need to be informed more important as compared to surgical patients’ parents (Wong, 1995).

### Reliability

In previous studies, Cronbach’s alpha coefficient was found ranging 0.69–0.95 for the entire inventory (Bandari et al., 2014; Coutu-Wakulczyk & Chartier, 1990; Sungeun, 2008; Toros, 2003; Unver, 1998) and 0.61–0.94 for its subscales (Bandari et al., 2014; Bijttebier et al., 2000; Chien, Ip & Lee, 2005; Leske, 1991; Sungeun, 2008). In this study, reliability estimates between each item and the entire inventory, as well as Cronbach’s alpha, indicated that the inventory and its subscales had good internal consistency. Item-total correlation coefficients also supported internal consistency.

### Strengths and limitations

This study’s strength is its methodology for adaptation. However, the fact that the sample is not representative for Turkey limits the findings’ generalizability. In addition, patients’ relatives whose patients’ duration of stay in ICU is 24 h may limit the results as 24 h could be insufficient to experience the ICU environment. Also, whether patient relatives have previous ICU experience or not was not asked, and that might limit the instrument’s discrimination strength.

## Conclusion

Understanding the needs of critical care patients’ relatives is important to create environments and organize services required for exercising patient rights, especially being appropriately informed. Meeting these needs would also increase patients and their relatives’ satisfaction with healthcare and caregivers. In that sense, CCFNI is a valuable inventory, and this study provides a reliable, valid adaptation of it. Its psychometric properties are acceptable. Furthermore, the validity and reliability of the subscales that measure different needs dimensions were established. The inventory’s Turkish version could be useful for helping healthcare workers provide services in a holistic approach and for policymakers to improve the quality of service. Finally, this version makes it possible to conduct studies for comparison to see how the inventory was structured in varied populations.

## Supplemental Information

10.7717/peerj.1208/supp-1 Supplemental Information 1 The CCFNI—Turkish version

10.7717/peerj.1208/supp-2 Supplemental Information 2 Raw data Raw data of the study

## References

[ref-1] Ægisdóttir S, Gerstein LH, Çinarbaş DC (2008). Methodological issues in cross-cultural counseling research: equivalence, bias, and translations. The Counseling Psychologist.

[ref-2] Artino AR, La Rochelle JS, Dezee KJ, Gehlbach H (2014). Developing questionnaires for educational research: AMEE Guide No. 87. Medical Teacher.

[ref-3] Bandari R, Heravi-Karimooi M, Rejeh N, Montazeri A, Zayeri F, Mirmohammadkhani M, Vaismoradi M (2014). Psychometric properties of the Persian version of the Critical Care Family Needs Inventory. Journal of Nursing Research.

[ref-4] Bijttebier P, Delva D, Vanoost S, Bobbaers H, Lauwers P, Vertommen H (2000). Reliability and validity of the Critical Care Family Needs Inventory in a Dutch-speaking Belgian sample. Heart and Lung.

[ref-5] Buyukozturk S (2004). Handbook of data analysis in social sciences (In Turkish).

[ref-6] Chien WT, Ip WY, Lee IY (2005). Psychometric properties of a Chinese version of the Critical Care Family Needs Inventory. Research in Nursing and Health.

[ref-7] Cohen J (1988). Statistical power analysis for the behavioural sciences.

[ref-8] Cokluk O, Sekercioglu G, Buyukozturk S (2010). Multivariable statistics for social sciences (In Turkish).

[ref-9] Coutu-Wakulczyk G, Chartier L (1990). French validation of the Critical Care Family Needs Inventory. Heart and Lung.

[ref-10] Curry S (1995). Identifying family needs and stresses in the intensive care unit. British Journal of Nursing.

[ref-11] Gjersing L, Caplehorn J, Clausen T (2010). Cross-cultural adaptation of research instruments: language, setting, time and statistical considerations. BMC Medical Research Methodology.

[ref-12] Gozum S, Aksayan S (2003). A guide for transcultural adaptation of the scale II: psychometric characteristics and cross-cultural comparison (In Turkish). Hemşirelikte Araştırma Geliştirme Dergisi.

[ref-13] Johnson SK, Craft M, Titler M, Halm M, Kleiber C, Montgomery LA, Megivern K, Nicholson A, Buckwalter K (1995). Perceived changes in adult family members’ roles and responsibilities during critical illness. Image: the Journal of Nursing Scholarship.

[ref-14] Kleinpell RM, Powers MJ (1992). Needs of family members of intensive care unit patients. Applied Nursing Research.

[ref-15] Kutlu Y (2000). Problems of family members of patients staying in intensive care units (In Turkish). Yoğun Bakım Hemşireliği Dergisi.

[ref-16] Lawshe CH (1975). A quantitative approach to content validity. Personnel Psychology.

[ref-17] Lee LY, Lau YL (2003). Immediate needs of adult family members of adult intensive care patients in Hong Kong. Journal of Clinical Nursing.

[ref-18] Leske JS (1986). Needs of relatives of critically ill patients: a follow-up. Heart and Lung.

[ref-19] Leske JS (1991). Internal psychometric properties of the Critical Care Family Needs Inventory. Heart and Lung.

[ref-20] Leung KK, Chien WT, Mackenzie AE (2000). Needs of Chinese families of critically ill patients. Western Journal of Nursing Research.

[ref-21] Macey BA, Bouman CC (1991). An evaluation of validity, reliability, and readability of the Critical Care Family Needs Inventory. Heart and Lung.

[ref-22] McKenzie JF, Wood ML, Kotecki JE, Clark JK, Brey RA (1999). Establishing content validity: using qualitative and quantitative steps. American Journal of Health Behavior.

[ref-23] Mendonca D, Warren NA (1998). Perceived and unmet needs of critical care family members. Critical Care Nursing Quarterly.

[ref-24] Molter NC (1979). Needs of relatives of critically ill patients: a descriptive study. Heart and Lung.

[ref-25] O’Malley P, Favaloro R, Anderson B, Anderson ML, Siewe S, Benson-Landau M, Deane D, Feeney J, Gmeiner J, Keefer N, Mains J, Riddle K (1991). Critical care nurse perceptions of family needs. Heart and Lung.

[ref-26] Ozgursoy BN, Akyol DA (2008). Needs of family members of patients in intensive care unit (In Turkish). Yoğun Bakım Hemşireliği Dergisi.

[ref-27] Price DM, Forrester DA, Murphy PA, Monaghan JF (1991). Critical care family needs in an urban teaching medical center. Heart and Lung.

[ref-28] Quinn S, Redmond K, Begley C (1996). The needs of relative visiting adult critical care units as perceived by relatives and nurses. Part I. Intensive & Critical Care Nursing.

[ref-29] Rukholm E, Bailey P, Coutu-Wakulczyk G, Bailey WB (1991). Needs and anxiety levels in relatives of intensive care unit patients. Journal of Advanced Nursing.

[ref-30] Siddiqui S, Sheikh F, Kamal R (2011). What families want—an assessment of family expectations in the ICU. International Archives of Medicine.

[ref-31] Sousa VD, Rojjanasrirat W (2011). Translation, adaptation and validation of instruments or scales for use in cross-cultural health care research: a clear and user-friendly guideline. Journal of Evaluation in Clinical Practice.

[ref-32] Sungeun Y (2008). A mixed methods study on the needs of Korean families in the intensive care unit. Australian Journal of Advanced Nursing.

[ref-33] Tekinsoy P (2005). Yogun bakim unitesinde tedavi goren hastalarin refakatcilerinin gereksinimlerinin saptanmasi. Thesis for a Master of Arts degree (In Turkish).

[ref-34] Toros F (2003). Yoğun bakım ünitesinde hastası olan aile üyelerinin gereksinimlerinin karşılanması(Meeting the needs of family members of patients in ICUs). MA Thesis.

[ref-35] Unver Y (1998). Yoğun Bakım Ünitesinde Yatan Hasta Ailelerinin Gereksinimlerinin Saptanması ve Ailenin Bakıma Katılım Düzeyinin İncelenmesi (Meeting the needs of family members of patients in ICUs and examining the level of family participation to patient care). MA Thesis.

[ref-36] Wong F (1995). The needs of families of critically ill patients in a Chinese community. Hong Kong Nursing Journal.

