# Supplementary material for: Adaptation of the Critical Care Family Need Inventory to the Turkish population and its psychometric properties
Source: PeerJ. 2015 Aug 20;3:e1208. doi: 10.7717/peerj.1208 (PMC4563234; doi:10.7717/peerj.1208)
Supplement: Supplemental Information 1 [file peerj-03-1208-s001.pdf]

Zor olan bu süreçte aşağıdaki ihtiyaçların sizin için *ne kadar önemli olduğunu* **işaretleyiniz.**

1- Hastamla ilgili beklenen sonucu bilmek

☐ Önemsiz ☐ Az önemli ☐ Önemli ☐ Çok önemli

2- Hastamı ilk ziyaretimden önce karşılaşacağım yoğun bakım ortamının anlatılması

☐ Önemsiz ☐ Az önemli ☐ Önemli ☐ Çok önemli

3- Hastamın doktoruyla her gün görüşebilmek

☐ Önemsiz ☐ Az önemli ☐ Önemli ☐ Çok önemli

4- Hastamı ziyarete gelemediğimde telefonla bilgi alabilmek

☐Önemsiz ☐Az önemli ☐Önemli ☐Çok önemli

5- Sorularımın gerçekçi bir şekilde cevaplanması

☐Önemsiz ☐Az önemli ☐Önemli ☐Çok önemli

6- Özel durumlarda ziyaret saatlerinin değiştirilebilmesi

☐Önemsiz ☐Az önemli ☐Önemli ☐Çok önemli

7- Yaşadığım durumla ilgili duygularım hakkında birileriyle konuşabilmek

☐Önemsiz ☐Az önemli ☐Önemli ☐Çok önemli

8- Hastanede iyi yemek yiyebileceğim bir yerin olması

☐Önemsiz ☐Az önemli ☐Önemli ☐Çok önemli

9- Hastamı ziyaret ederken ne yapılacağını belirten bir liste verilmesi

☐Önemsiz ☐Az önemli ☐Önemli ☐Çok önemli

10- Her istediğimde hastamı ziyaret edebilmek

☐Önemsiz ☐Az önemli ☐Önemli ☐Çok önemli

11- Yoğun bakımdaki hangi personelden (hekim/hemşire/sekreter) ne tür bilgi alabileceğimi bilmek

☐Önemsiz ☐Az önemli ☐Önemli ☐Çok önemli

12- Arkadaş ya da akrabalarımın bana destek olmak için yanımda olması

☐Önemsiz ☐Az önemli ☐Önemli ☐Çok önemli

13- Hastama uygulanan müdahalelerin neden yapıldığını bilmek

☐Önemsiz ☐Az önemli ☐Önemli ☐Çok önemli

14- Hastam için umut olduğunu hissetmek

☐Önemsiz ☐Az önemli ☐Önemli ☐Çok önemli

15- Hastama hangi sağlık personelinin sağlık hizmeti sunduğunu bilmek

☐Önemsiz ☐Az önemli ☐Önemli ☐Çok önemli

16- Hastama nasıl bir tıbbi tedavi uygulandığını bilmek

☐Önemsiz ☐Az önemli ☐Önemli ☐Çok önemli

17- Hastama mümkün olan en iyi tedavinin verildiğinden emin olmak

☐Önemsiz ☐Az önemli ☐Önemli ☐Çok önemli

18- Hastanede yalnız kalabileceğim bir yerin bulunması

☐Önemsiz ☐Az önemli ☐Önemli ☐Çok önemli

19- Hastama ne yapıldığını tam olarak bilmek

☐Önemsiz ☐Az önemli ☐Önemli ☐Çok önemli

20- Bekleme odasında rahat koltukların olması

☐Önemsiz ☐Az önemli ☐Önemli ☐Çok önemli

21- Hastane personeli tarafından anlayışla karşılanma

☐Önemsiz ☐Az önemli ☐Önemli ☐Çok önemli

22- Hastane masrafları ile ilgili sorunlarıma yardım edebilecek birisinin olması

☐Önemsiz ☐Az önemli ☐Önemli ☐Çok önemli

23- Bekleme odası yakınında kullanabileceğim bir telefonun bulunması

☐Önemsiz ☐Az önemli ☐Önemli ☐Çok önemli

24- Hastanede bir din görevlisine ulaşabilmek

☐Önemsiz ☐Az önemli ☐Önemli ☐Çok önemli

25- Hastamın ölüm olasılığı hakkında konuşabilmek

☐Önemsiz ☐Az önemli ☐Önemli ☐Çok önemli

26- Yoğun bakımda hastamı ziyaret ederken benimle birlikte başka bir yakınımın daha olması

☐Önemsiz ☐Az önemli ☐Önemli ☐Çok önemli

27- Yanımda benim sağlığımla ilgilenecek birinin daha olması

☐Önemsiz ☐Az önemli ☐Önemli ☐Çok önemli

28- Hastaneden ayrıldığım zaman gözümün arkada kalmaması

☐Önemsiz ☐Az önemli ☐Önemli ☐Çok önemli

29- Her gün aynı hemşireyle görüşebilmek

☐Önemsiz ☐Az önemli ☐Önemli ☐Çok önemli

30- İstediğim zaman çekinmeden ağlayabileceğimi hissetmek

☐Önemsiz ☐Az önemli ☐Önemli ☐Çok önemli

31- Bana, sorunlarımın çözümünde yardımcı olabilecek kişiler önerilmesi

☐Önemsiz ☐Az önemli ☐Önemli ☐Çok önemli

32- Bekleme odası yakınında hasta yakınlarının kullanabileceği bir tuvalet olması

☐Önemsiz ☐Az önemli ☐Önemli ☐Çok önemli

33- İstediğim zaman yalnız kalabilmek

☐Önemsiz ☐Az önemli ☐Önemli ☐Çok önemli

34- Ailevi sorunlarımın çözümünde yardımcı olabilecek kişilere yönlendirilmek

☐Önemsiz ☐Az önemli ☐Önemli ☐Çok önemli

35- Verilen bilgilerin anlaşılır olması

☐Önemsiz ☐Az önemli ☐Önemli ☐Çok önemli

36- Ziyaret saatlerinin zamanında başlaması

☐Önemsiz ☐Az önemli ☐Önemli ☐Çok önemli

37- Dini ibadetimi nerede yapabileceğim hakkında bilgi verilmesi

☐Önemsiz ☐Az önemli ☐Önemli ☐Çok önemli

38- Hastamın bakımına yardım etmeme izin verilmesi

☐Önemsiz ☐Az önemli ☐Önemli ☐Çok önemli

39- Hastamın başka bir yere nakli planlanıyorsa fikrimin alınması

☐Önemsiz ☐Az önemli ☐Önemli ☐Çok önemli

40- Ben evdeyken hastamın durumunda değişiklik olduğunda haber verilmesi

☐Önemsiz ☐Az önemli ☐Önemli ☐Çok önemli

41- Günde en az bir kere hastam hakkında bilgi almak

☐Önemsiz ☐Az önemli ☐Önemli ☐Çok önemli

42- Hastane personelinin hastamla ilgilendiğinden emin olmak

☐Önemsiz ☐Az önemli ☐Önemli ☐Çok önemli

43- Hastamın durumuyla ilgili gelişmeleri bilmek

☐Önemsiz ☐Az önemli ☐Önemli ☐Çok önemli

44- Hastamı sık sık görebilmek

☐Önemsiz ☐Az önemli ☐Önemli ☐Çok önemli

45- Bekleme odasının yoğun bakım birimine yakın olması

☐Önemsiz ☐Az önemli ☐Önemli ☐Çok önemli

Yukarıda değinilmeyen fakat sizin eklemek istediğiniz başka ihtiyaçlarınız varsa, lütfen aşağıya yazınız:

1-…………………………………………………………………………………...

2-…………………………………………………………………………………...

3-…………………………………………………………………………………...

4…………………………………………………………………………………...
